# 液体活检及其在非小细胞肺癌诊治中的研究进展

**DOI:** 10.3779/j.issn.1009-3419.2016.06.19

**Published:** 2016-06-20

**Authors:** 迪凡 郑, 海泉 陈

**Affiliations:** 200032 上海，复旦大学附属肿瘤医院胸外科 Department of Thoracic Surgery, Fudan University Shanghai Cancer Center, Shanghai 200032, China

**Keywords:** 液体活检, 循环肿瘤细胞, 循环肿瘤DNA, 肺肿瘤, Liquid biopsy, Circulating tumor cells, Circulating tumor DNA, Lung neoplasms

## Abstract

随着近几年科学技术的进步，液体活检技术也有了长足的发展，并在肿瘤的早期诊断及后期治疗中扮演着越来越重要的角色。相比于传统的组织活检，液体活检以其独有的无创性、便捷性、高重复性等特点在临床上得到更多的青睐，在未来有着巨大的发展潜力。本文重点探讨了循环肿瘤细胞（circulating tumor cells, CTCs）和循环肿瘤DNA（circulating tumor DNA, ctDNA），作为液体活检最重要的两个检测对象，其历史、生物学特性，检测手段，局限性及其在非小细胞肺癌诊治中的应用。

## Abstract (en)

With the advances of technology, great progresses have been made in liquid biopsy in recent years. Liquid biopsy is currently playing a more and more important role in early diagnosis and treatment of cancer. Compared with traditional tissue biopsy, liquid biopsy is more popular in clinical practice due to its non-invasiveness, convenience and high repeatability. It has huge potential in the future. This review introduces circulating tumor cells (CTCs) and circulating tumor DNA (ctDNA) as the most important objects in liquid biopsy, mainly focusing on their history, biological characteristics, detection technologies, limitations and applications in non-small cell lung cancer.

随着近几年科学技术的进步，尤其是细胞分离技术和测序技术的发展，液体活检，在肿瘤的精准治疗中日益重要。恶性肿瘤的精确诊断与治疗离不开肿瘤组织的获取及后续的病理学、分子生物学检测。肿瘤早期患者的手术切除及肿瘤中晚期患者或不适宜手术患者的诊断性组织活检是获取肿瘤组织的主要手段。近年来，肿瘤患者的生存时间日益延长，生存质量不断提高也得益于各类最新的筛查活检技术。这些技术的广泛普及使得更多的早期患者能更早地进行根治性手术治疗。然而，组织活检限于肿瘤生长部位、取材大小等因素，并不能百分之百发现恶性肿瘤细胞^[1, 2]^；同时，肿瘤异质性使得单次组织活检、基因检测并不能完整描述肿瘤整体在基因层面上的改变^[3]^。任何肉眼可见的肿瘤均提示患者并非在疾病的最早期。对于恶性肿瘤精确诊治的要求不断提高，液体活检正逐渐展露其优势。液体活检是一种从血液等非实性生物组织中取样并分析，主要用于诊断或监测肿瘤疾病的方法^[4]^。血液中的循环肿瘤细胞（circulating tumor cells, CTC）和循环肿瘤DNA（circulating tumor DNA, ctDNA）和肿瘤直接相关，最大限度覆盖了患者疾病的特点。本文着重介绍CTC和循环肿瘤DNA及其在非小细胞肺癌（non-small cell lung cancer, NSCLC）诊治中的应用。

## 1 CTC和ctDNA

CTC是指由肿瘤原发灶或转移灶进入血液循环的肿瘤细胞^[5]^。1869年，澳大利亚医生在一位死去的癌症患者的血液中发现了和肿瘤细胞形态一致的细胞，并提出了"血液中的肿瘤细胞可能揭示出患者原发肿瘤的生物特征"这一论断^[6]^。人体1 mL血液中含有大约5×10^9^个红细胞，7×10^6^个白细胞，而即使在晚期肿瘤患者的血液中也只有1个-10个CTC^[7]^。CTC含量极微，临床现有的技术手段无法进行有效的筛选和分析。组织中的肿瘤细胞进入血液循环的机制并不十分明确。在乳腺癌患者行根治性手术多年后血液中依然能够检测到CTC，提示CTC不仅来源于肿瘤原发灶，还可来源于肿瘤转移灶或微转移灶^[8]^。CTC在血液中的半衰期很短，只有几个小时^[8]^，若无及时补充，在24小时之后，血液中的CTC在现有技术条件下将无法检测到^[9]^。CTC在血液中既有单个存在，也有多个聚集成簇存在，极少部分在发现时处于有丝分裂状态。镜下CTC的形态也各有差异，部分细胞形态和原发部位肿瘤细胞的形态相似，部分细胞表现为凋亡的状态，还有部分细胞残缺不全^[10]^。CTC在基因突变，拷贝数变异，及不同信号通路传递的强度上与原发灶或转移灶细胞既有相似之处，又有自身独立的特征^[10]^。

循环游离DNA（circulating free DNA, cfDNA）是释放入血液中的被降解的DNA片段^[11]^，最早在1948年被发现^[12]^。血液中的游离DNA片段一部分的来自正常细胞，另一部分来自肿瘤细胞，被称为ctDNA。根据肿瘤患者的分期，肿瘤负荷的不同，ctDNA占所有循环游离DNA的比例在1%到93%之间波动^[13, 14]^。ctDNA大部分来自凋亡或坏死的肿瘤细胞^[14, 15]^。之前有猜测ctDNA可能大部分来源于CTC，然而对比血液中ctDNA的含量和CTC的浓度可以发现，在中晚期肿瘤患者的每毫升血液中平均含15 ng ctDNA，而一个细胞含有约6 pg DNA，则推算出至少每毫升血液中存在上千个CTC，然而事实上，平均每毫升血液只含CTC 1个-10个。肿瘤患者的血液中循环DNA的含量要高于正常人。血液中游离DNA的片段长度集中在180 bp到200 bp之间，片段长度提示这些DNA主要由细胞凋亡产生^[14]^。在大多数的进展期肿瘤如胰腺癌、卵巢癌、结直肠癌、膀胱癌等患者血液中都可以检测到ctDNA，在局限期肿瘤患者中，也有将近一半比例的患者能够检测到CTC。对于那些无法检测到CTC的患者，其中的大部分能够检测到ctDNA^[15]^。根据实验推算，ctDNA的半衰期在2小时左右^[13]^。有研究^[16]^发现，细胞可以摄取血液中的ctDNA，并且ctDNA在体外实验中能够成功诱导正常3T3细胞向肿瘤细胞转化。如果这一结论能够进一步在人体中被验证，即可说明肿瘤细胞能够主动向外界释放肿瘤DNA，并通过肿瘤DNA将正常细胞"肿瘤化"。那么，ctDNA，将不仅仅只是一种被动监测的标志物，而是一种特殊形式的必须要主动消灭的肿瘤。

## 2 液体活检检测手段

由于CTC在人体血液中数量极少，绝大部分的肿瘤患者，即使在晚期，每毫升血液也只有1个-10个CTC。检测的第一步即是要分离CTC。来源于上皮的CTC由于体积大于正常的血细胞，并且不易变形^[17, 18]^，可以用物理的手段来过滤掉较小的正常细胞，筛选出肿瘤细胞。通过设计适合孔径的筛板，能够获取到尽可能多的CTC。然而，并非所有的肿瘤细胞都在体积上大于正常血细胞，另有一部分肿瘤细胞具有向间质细胞转化的能力，同时也有变形能力，这些不确定因素都会降低物理筛选的效果。其他基于物理的筛选方法，如密度梯度离心法^[19]^、介电泳法^[20]^等也被用于筛选CTC。

最常用的CTC分离技术是抗体介导的肿瘤细胞捕获技术。用结合了磁珠的抗体蛋白去靶向肿瘤细胞，抗体多是上皮细胞表面所具有的蛋白标志物，再通过磁场导向，筛选出被抗体识别的细胞^[21, 22]^。还有一种反向选择的方法，即靶向红细胞和白细胞表面的蛋白标志物，筛选并分离去除，那剩下的大部分就是CTC^[23, 24]^。筛选出的疑似CTC还需进一步验证，如通过一些肿瘤相关标志物的抗体，肿瘤相关基因的PCR扩增，对获取的细胞进行细胞培养，并功能验证，还可以将细胞注入裸鼠体内检验成瘤性等^[25, 26]^。

获取到的CTC，在细胞和分子层面上，可以做和手术切除或活检得到的肿瘤组织一样的研究，如基因测序、基因表达分析、FISH、细胞免疫染色、细胞培养等^[27]^。

ctDNA与循环游离DNA的本质区别在于，ctDNA和肿瘤细胞中的DNA一样，存在基因突变，基因拷贝数变异等正常细胞不具有的基因层面改变，这也是区分并检测ctDNA的基础。由于ctDNA在血液中的含量极低，并且ctDNA占循环游离DNA的比例往往不高，传统的一代测序技术几乎不能检测到ctDNA，只有在极少数肿瘤晚期，且肿瘤负荷较大的患者身上，才会有阳性发现。

近几年技术手段的突飞猛进，包括数字PRC技术^[28, 29]^、BEAMing（beads, emulsion, amplification, and magnetics）法^[30, 31]^、PAP法^[32]^等用于检测ctDNA。其中BEAMing法能够检测比例在1:10, 000以下的基因突变，具有足够高的敏感性。并已经用于临床试验^[33]^。二代测序技术也越来越多地被用于检测ctDNA，兼具极高的敏感性和特异性，还能够获得所有ctDNA的序列信息^[34, 35]^。

## 3 液体活检的优势与局限性

医学技术的不断发展使得肿瘤治疗手段日益多元化。每一个肿瘤患者，想要获得最佳的治疗策略，不仅需要临床资料，更重要的是肿瘤在组织层面，细胞层面及分子层面的全方位的信息。通过手术切除或诊断性组织活检的方法获得的肿瘤组织在一定程度上能够提供上述信息。但是肿瘤的异质性使得一次的检测结果并不足以揭示出肿瘤基因图谱的全貌，以偏概全的结论还有可能误导治疗方案^[3, 36]^。对于晚期患者和不适宜手术患者，部分区域的肿瘤很难进行取样。随着时间推移和治疗的影响，肿瘤的基因图谱也随之改变，再次或多次对患者进行活检取样在临床上亦不现实。液体活检技术仅通过抽取患者外周血液进行检测分析并获得患者肿瘤相关信息。对患者不造成创伤，操作方便快捷，并能够反复获取，易于实时监控。从血液中获得CTC和ctDNA由其本身的生物学特性（半衰期都在24小时之内）必定是最新鲜、实时的，并且CTC，ctDNA可来源于肿瘤组织的任何部分，其中包含了异质性的各个方面，能够反映肿瘤的全貌。CTCctDNA携带的信息各有侧重。ctDNA相比CTC更实时，更能动态反映人体内肿瘤的变化情况^[37, 38]^，而CTC不仅包含DNA信息，还有RNA、蛋白质信息等，更能全方位揭示肿瘤特征。两者相互结合，意义更大。

液体活检技术目前也存在局限性。无论CTC还是ctDNA在血液中都极其微量，需要技术的不断革新来增加仪器检测的灵敏度。新技术的使用成本较高，应用范围不广，从实验室到临床上大规模应用还有很长一段路要走。每一项液体活检检测技术的敏感性与特异性如何？液体活检在肿瘤的早期诊断中究竟能达到多早？液体活检与金标准病理组织活检的相关性有多高？液体活检揭示肿瘤动态变化的反应有多灵敏？这些都是液体活检技术亟待解决的理论问题。最近有研究显示，针对单个CTC进行基因分析发现，同一个体中，不同的CTC在基因突变或是信号通路上也存在差异^[39]^。液体活检是否能够真正克服肿瘤异质性问题还需进一步探索。

## 4 液体活检在非小细胞肺癌中的应用

液体活检是早期诊断非小细胞肺癌的有效手段。有研究以无临床肿瘤证据的慢性阻塞性肺病（chronic obstructive pulmonary disease, COPD）患者为目标人群，检测他们血液中是否存在CTC，结果显示有3%的个体检测到了CTC，并且在1到4年时间内均出现了计算机断层扫描（computed tomography, CT）可见的肺结节，并手术证实为早期肺癌^[40]^。液体活检能够检测非小细胞肺癌患者肿瘤中所有的基因异常。对血液中的ctDNA进行深度测序能够检测出迄今为止报道的所有基因点突变，单核苷酸变异和融合基因，并具有极高的敏感度（85%）和特异度（96%）^[41]^。在ctDNA中检测表皮生长因子受体（epidermal growth factor receptor, *EGFR*）突变情况与在切除的肿瘤组织中检测间有较高的符合率^[42]^。对CTC采用过滤器相适应的荧光原位杂交技术（FA-FISH）检测出融合基因（如*ALK*），检测结果和肿瘤活检一致。这不仅能作为融合基因诊断工具，同时也还能作为融合基因靶向治疗疗效的监测工具^[43]^。

液体活检能动态监测非小细胞肺癌患者对治疗的反应情况。ctDNA的水平和患者体内肿瘤负荷显著相关，且比影像学检查更早提示患者对治疗的反应^[41]^。治疗过程中，连续地监测CTC的浓度变化和CTC中DNA的基因突变情况，当患者对治疗反应良好时，CTC浓度呈下降趋势；相反，肿瘤进展时，CTC浓度不断上升，并且CTC中新的基因突变形式也不断出现。CTC这一系列变化情况和随后的影像学检查结果相符^[44, 45]^。

液体活检可以及时反映非小细胞肺癌患者对靶向治疗的耐药情况。具有*EGFR*基因突变的非小细胞肺癌患者，可以使用EGFR抑制剂进行治疗。然而治疗一段时间后部分患者会产生耐药性，继发的EGFR-T790M突变是最常见的耐药原因^[46]^。通过检测靶向治疗患者的ctDNA可以发现，一半以上的出现耐药患者的ctDNA中可以检测到*EGFR*-T790M突变，而未经靶向治疗患者的ctDNA中没有*EGFR*-T790M突变。这说明ctDNA能比较准确的反映非小细胞肺癌患者对靶向治疗的耐药情况。同时，ctDNA中*EGFR*-T790M突变的浓度变化还可以用于判断患者耐药强度的变化情况^[47, 48]^。此外，运用液体活检技术，检测CTC的动态变化情况短期内可以评估根治性手术后患者体内肿瘤的残余情况。长期可以更早的发现患者肿瘤的复发情况^[8]^。

## 5 展望

液体活检具有独特的技术优势和广泛的应用前景，有着广阔的发展空间。然而，现阶段液体活检还不能够取代组织活检的金标准地位。液体活检所提供的信息也还需要更先进的技术来做出更深层次的解读。

液体活检是一个不断发展的概念，随时间推移，其外延也在不断扩展。从广为人知的蛋白类肿瘤标志物，到如今的CTC、ctDNA，再到最近几年兴起的外泌体^[49]^，液体活检始终是一个试验性、探索性的课题。相信在不久的将来，液体活检将能够更有效地促进非小细胞肺癌患者的早期诊断及后期治疗，为广大患者带来福音。
